# Complicaciones hospitalarias en una unidad geriátrica de agudos

**DOI:** 10.7705/biomedica.5664

**Published:** 2021-06-15

**Authors:** David José Coca, Sandra Milena Castelblanco, Diego Andrés Chavarro-Carvajal, Luis Carlos Venegas-Sanabria

**Affiliations:** 1 Unidad de Geriatría, Hospital Universitario San Ignacio, Bogotá, D.C., Colombia Unidad de Geriatría Hospital Universitario San Ignacio BogotáD.C. Colombia; 2 Centro de Memoria y Cognición Intellectus, Hospital Universitario San Ignacio, Bogotá, D.C., Colombia Centro de Memoria y Cognición Intellectus Hospital Universitario San Ignacio BogotáD.C. Colombia; 3 Instituto de Envejecimiento, Facultad de Medicina, Pontificia Universidad Javeriana, Bogotá, D.C., Colombia Pontificia Universidad Javeriana Instituto de Envejecimiento Facultad de Medicina Pontificia Universidad Javeriana BogotáD.C. Colombia

**Keywords:** geriatría, envejecimiento, hospitalización/complicaciones, desnutrición, Geriatrics, aging, hospitalization/complications, malnutrition

## Abstract

**Introducción.:**

Las complicaciones hospitalarias ocurren con gran frecuencia en personas mayores de 65 años hospitalizadas y conllevan peores resultados clínicos. Son pocos los estudios sobre los factores asociados con las complicaciones hospitalarias en la atención de adultos mayores.

**Objetivo.:**

Evaluar los factores asociados con las complicaciones hospitalarias en una unidad geriátrica de agudos en Bogotá, Colombia.

**Materiales y métodos.:**

Se hizo un estudio observacional analítico basado en una cohorte retrospectiva que incluyó 1.657 pacientes mayores de 65 años atendidos en una unidad de hospitalización en Bogotá, Colombia. La variable dependiente fueron las complicaciones hospitalarias y, las independientes, la dependencia funcional, la demencia, el estado nutricional, el soporte social, las comorbilidades y la polifarmacia. Se utilizó el modelo de regresión lineal de Poisson para determinar las variables asociadas.

**Resultados.:**

En el análisis bivariado se encontró que la dependencia funcional (razón de prevalencia, RP=2,092; p≤0,001) y la malnutrición (RP=2,850; p≤0,001) eran factores asociados con una mayor tasa de complicaciones hospitalarias. En el análisis multivariado aparecían como factores independientes (dependencia funcional: RP=1,931 y p=0,003; malnutrición: RP=2,502 y p=0,002).

**Conclusión.:**

El hacer una evaluación integral que permita determinar la dependencia funcional y la malnutrición en el momento de ingreso en las unidades de hospitalización, permitiría predecir complicaciones hospitalarias.

## Abstract (en)

**Introduction:**

In-hospital complications frequently occur in hospitalized people over 65 worsening their clinical outcomes. There are, however, few studies on the factors associated with in-hospital complications in elderly patient care.

**Objective:**

To evaluate factors associated with in-hospital complications in a geriatric acute care unit in Bogotá, Colombia

**Materials and methods:**

We conducted an analytical, observational, retrospective study in a cohort of 1,657 patients over 65 years of age who received care in the geriatric unit of a high complexity hospital in Bogotá, Colombia. The dependent variable was in-hospital complications and the independent variables, the degree of functional dependence on admission, dementia, nutritional status, social support, comorbidity, and polypharmacy. We used Poisson’s linear regression model to identify associated variables.

**Results:**

The bivariate analysis showed that functional dependence (PR=2.092, p≤0.001) and malnutrition (PR=2.850, p≤0.001) were associated with a higher rate of hospital-acquired infection. In the multivariate analysis, functional dependence (PR=1.931, p=0.003) and malnutrition (PR=2.502, p=0.002) remained independent factors for in-hospital complications.

**Conclusion:**

In acute care centers, integral assessment at admission to identify functional dependence and malnutrition predicts in-hospital complications.

Las complicaciones que se presentan durante la hospitalización de los mayores de 65 años, constituyen situaciones clínicas adversas que afectan a los pacientes durante la admisión o a lo largo de la hospitalización ^1^. Las más comunes son las infecciones hospitalarias, los problemas relacionados con los tratamientos y las condiciones trombóticas, las cuales constituyen marcadores de la imposibilidad de responder a las necesidades básicas de salud de los pacientes. Estas complicaciones provocan un mayor deterioro clínico y funcional, prolongan la hospitalización, favorecen la instauración de comorbilidades (permanentes o temporales) y aumentan el riesgo de mortalidad ^1^.

En múltiples estudios en Europa y en los Estados Unidos, se ha establecido que factores como la infección hospitalaria, el delirio (síndrome confusional agudo), el deterioro funcional hospitalario y las lesiones de piel por presión, entre otros, predisponen a peores resultados hospitalarios y, por consiguiente, a prolongar la estancia hospitalaria y al aumento de la mortalidad ^2^^-^^4^. Diversos factores pueden asociarse con una mayor frecuencia de complicaciones hospitalarias y algunos ya han sido claramente descritos, como la malnutrición, el deterioro cognitivo, la prescripción inapropiada de medicamentos y el detrimento del estado funcional ^4^.

Al igual que en otros países latinoamericanos, en Colombia, 8 de cada 10 adultos mayores padece más de una condición crónica ^2^ y, cuando presentan enfermedades agudas o descompensación de las crónicas, deben ser hospitalizados en unidades de atención convencional, ya que muy pocos centros asistenciales cuentan con unidades de geriatría ^5^. Esto provoca un incremento de los costos de atención y un mayor tiempo de hospitalización ^6^.

En un estudio en Perú, se determinó que comorbilidades como el cáncer, la enfermedad pulmonar, la enfermedad cardiovascular y el deterioro del estado funcional, se asociaban con la aparición de complicaciones hospitalarias ^7^. Sin embargo, aún no hay estudios que describan claramente las complicaciones hospitalarias y los factores asociados en esta población en Colombia.

En el presente estudio, se evaluó una muestra de adultos mayores atendidos en el Hospital Universitario San Ignacio que presentaron complicaciones hospitalarias. Se utilizaron instrumentos de evaluación estandarizados para determinar dichos factores y su asociación.

## Materiales y métodos

### Diseño

Se llevó a cabo un estudio retrospectivo observacional analítico basado en una cohorte de la población atendida en el ámbito hospitalario entre el 1° de diciembre de 2015 y el 31 de junio de 2018.

Los datos obtenidos mediante la revisión de las historias clínicas se consignaron en una base de datos con acceso únicamente para los investigadores, construida con base en las variables sociodemográficas, el estado funcional, la demencia, la malnutrición, la polifarmacia, las comorbilidades, el soporte social y las complicaciones hospitalarias.

### Población

Se incluyeron adultos mayores de 65 años hospitalizados en el Servicio de Geriatría del Hospital Universitario San Ignacio, a quienes se les hizo una valoración geriátrica integral. Se excluyeron a aquellos internados por enfermedades psiquiátricas, a los que no tenían los datos completos en la base de datos, a los que solicitaron retiro voluntario del hospital en contra de la recomendación médica y a los remitidos a otra institución.

La variable dependiente fueron las complicaciones hospitalarias, definidas como cualquier problema médico o resultado clínico inesperado durante el transcurso de la hospitalización, relacionado o no con el tratamiento ^8^. Entre ellas, se consideraron las infecciones adquiridas en el ámbito hospitalario detectadas 48 horas después del ingreso ^9^, la aparición de úlceras por presión 24 horas después del ingreso ^10^, las caídas o traumas durante la estancia, los eventos trombóticos documentados 48 horas después del ingreso (trombosis venosa profunda y tromboembolia pulmonar) y las reacciones adversas a los medicamentos ^1^^,^^7^

Las variables independientes incluyeron: la edad, como variable continua; el sexo (masculino o femenino); la dependencia moderada en las actividades básicas de la vida diaria, reflejada en un puntaje menor o igual a 60 en la escala de Barthel al ingreso; el diagnóstico de demencia al ingreso, como variable dicotómica (tenerlo o no) ^11^; la malnutrición, definida por un puntaje menor de 24 en el *Mini-Nutritional Assessment* (MNA) ^12^^,^^13^; y el poco soporte social, definido de forma dicotómica según si el paciente tenía o no cuidador en el momento de ingreso ^14^.

Además, el número de comorbilidades, considerando su distribución no normal y calculando la mediana para definirla como dicotómica si presentaba cinco comorbilidades o más, o menos de cinco (entre ellas, hipertensión arterial sistémica, falla cardiaca, diabetes mellitus, enfermedad renal crónica, neumopatía o enfermedad pulmonar obstructiva crónica, enfermedad cerebrovascular, hipotiroidismo, osteoartrosis y osteoporosis) ^15^; la polifarmacia, definida como variable dicotómica si el paciente tomaba 5 medicamentos o más en el momento de ingreso, o menos de 5 ^16^, y la enfermedad índice motivo de la hospitalización, es decir, la causa principal del ingreso del paciente ^17^.

### Análisis estadístico

Se hizo un análisis descriptivo con la información de las variables de interés, utilizando medidas de tendencia central y de dispersión. La media, la mediana, la desviación estándar y el rango intercuartílico (RIC) se ajustaron a los criterios de distribución normal o no normal, utilizando la prueba de Shapiro-Wilk. Las variables categóricas se presentan como tablas de frecuencia. En el análisis bivariado se aplicó un nivel de significación o valor de p menor de 0,05. En cuanto al análisis de las variables dicotómicas, se utilizó la prueba de ji al cuadrado y, en el de las variables continuas, la prueba U de Mann-Whitney, para verificar si había significación estadística.

Por último, para determinar los criterios de distribución normal o no normal, se utilizó la prueba de Shapiro-Wilk considerando las variables de edad, sexo, puntaje en la escala de Barthel en el ingreso, demencia, malnutrición, comorbilidades y polifarmacia. Se reportaron las razones de prevalencia (RP) obtenidas con intervalos de confianza del 95% (IC95%). El análisis de datos se hizo con el programa estadístico Stata™, versión 14,2 (College Station, TX, Estados Unidos).

### Consideraciones éticas

El estudio no entrañó riesgo según la Resolución 8430 del Ministerio de Salud y la Declaración de Helsinki, y fue aprobado por el Comité de Ética de la Pontificia Universidad Javeriana y el del Hospital Universitario San Ignacio en Bogotá (Colombia).

## Resultados

Se incluyeron 1.656 pacientes hospitalizados en la Unidad de Geriatría del Hospital de San Ignacio. La mediana de edad fue de 86 años (RIC=81-90); la mayoría de los pacientes (57,87%) era de sexo femenino y la prevalencia de complicaciones hospitalarias fue del 7,06%, y la infección hospitalaria fue la causa más frecuente de complicación (46,15%).

El 83,2% de los pacientes tenía síndromes geriátricos: 73,73%, malnutrición; 61,11%, algún tipo de demencia, más frecuentemente enfermedad de Alzheimer (35,42%); 60,32%, polifarmacia, y el 51,81%, un mayor grado de dependencia funcional. Se reportó poco soporte social en el 16,06% de los pacientes y un importante número de comorbilidades (5 o más) en el 51,77%.

Las principales causas de hospitalización fueron las infecciones (28,38%), seguidas de las afecciones cardiovasculares (25,18%), las respiratorias (14,25%) y las gastrointestinales (7,12%) (cuadro 1). La prevalencia de mortalidad fue del 37,67% en el grupo de pacientes que presentaron complicaciones hospitalarias y, de 8,58%, en el grupo que no las presentó, con una diferencia estadísticamente significativa (p<0,001).

**Cuadro 1:** Características de la población en estudio

Características de la población (N=1.656)					
	Sin complicación hospitalaria (n=1-539)		Con complicación hospitalaria (n=117)		p*
Variable	n o mediana	RIC o %	n o mediana	RIC o %	
Edad	86	P25 % 81 P75 % 90	86	P25 % 81 P75 % 90	0,321
Sexo					0,733
Masculino	646	92,68 %	51	7,32 %	
Femenino	893	93,12 %	66	6,88 %	
Dependencia moderada	777	90,56 %	81	9,44 %	<0,001
Demencia					0,607
No	599	93,45 %	42	6,55 %	
Sí	939	92,79 %	73	7,21 %	
Estado nutricional					<0,001
Adecuado	422	97,01 %	13	2,99 %	
Malnutrición	1.117	91,48 %	104	8,52 %	
Soporte social					0,796
Adecuado	1.290	93,01 %	97	6,99 %	
Poco soporte social	249	92,57 %	20	7,43 %	
Multimorbilidad					0,802
<5	742	93,10 %	55	6,90 %	
≥5	797	92,78 %	62	7,22 %	
Polifarmacia al ingreso					0,211
No	616	93,90 %	40	6,10 %	
Sí	922	92,29 %	77	7,71 %	
Enfermedad índice					0,004
Infecciosa	419	89,15 %	51	10,85 %	
Cardiovascular	395	94,72 %	22	5,28 %	
Respiratoria	227	96,19 %	9	3,81 %	
Gastrointestinal	106	90,60 %	11	9,40 %	
Mortalidad	132	8,58 %	44	37,61 %	<0,001

En el análisis bivariado, el mayor grado de dependencia funcional (RP=2,092; IC_95%_ 1,430-3,062; p≤0,001) y la malnutrición (RP=2,850; IC_95%_ 1,617-5,020; p≤0,001) fueron los factores asociados con una mayor posibilidad de complicaciones hospitalarias. La demencia, el poco soporte social, el mayor número de comorbilidades y tener polifarmacia al ingreso de la hospitalización, no fueron factores estadísticamente significativos (cuadro 2).

**Cuadro 2:** Análisis bivariado para evaluar los factores asociados con complicaciones hospitalarias

Análisis bivariado			
Variable	RP (IC_95%_)	p*
Edad	0,837 (0,590-1,189)	0,321
Sexo femenino	0,940 (0,661-1,337)	0,733
Dependencia moderada	2,092 (1,430-3,062)	<0,001
Demencia	1,100 (0,763-1,588)	0,607
Malnutrición	2,850 (1,617-5,020)	0,000
Poco soporte social	1,063 (0,668-1,689)	0,796
Cinco o más enfermedades	1,045 (0,736-1,484)	0,802
Polifarmacia al ingreso	1,264 (0,873-1,828)	0,213

* Modelo de regresión de Poisson

En el análisis multivariado con el modelo de regresión de Poisson, se evidenció que el mayor grado de dependencia funcional (RP=1,931; IC_95%_ 1,246-2,993; p=0,003) y la malnutrición (RP=2,502; IC_95%_ 1,396-4,486; p=0,002) continuaron siendo los factores asociados con una mayor posibilidad de complicaciones hospitalarias (cuadro 3).

**Cuadro 3:** Modelo de regresión logística para evaluar los factores asociados con complicaciones hospitalarias

Análisis multivariado			
	Bivariado	Multivariado	
Variable	RP (IC_95%_)	RP (IC_95%_)	p*
Edad	0,837 (0,590-1,189)	0,820 (0,578-1,164)	0,268
Sexo femenino	0,940 (0,661-1,337)	0,904 (0,636-1,286)	0,578
Dependencia moderada	2,092 (1,430-3,062)	1,931 (1,246-2,993)	0,003
Demencia	1,100 (0,763-1,588)	0,687 (0,458-1,030)	0,069
Malnutrición	2,850 (1,617-5,020)	2,502 (1,396-4,486)	0,002
Mal soporte social	1,063 (0,668-1,689)	0,999 (0,636-1,569)	0,999
Cinco o más enfermedades	1,045 (0,736-1,484)	0,934 (0,655-1,333)	0,709
Polifarmacia al ingreso	1,264 (0,873-1,828)	1,239 (0,843-1,820)	0,274

* Modelo de regresión de Poisson

## Discusión

En este estudio se analizaron los factores asociados con las complicaciones hospitalarias en una unidad geriátrica de agudos. Se incluyeron los datos de 1.656 sujetos mayores de 65 años que consultaron por diversas enfermedades: infecciosas, cardiovasculares, o pulmonares, o por descompensación de condiciones metabólicas. Se presentaron complicaciones durante la hospitalización en 117 (7,06%) sujetos, predominantemente en mujeres con malnutrición, múltiples enfermedades, compromiso cognitivo o polifarmacia. Además, se encontró que situaciones como la demencia, la dependencia funcional y la malnutrición fueron los principales factores asociados con la aparición de complicaciones hospitalarias en las personas mayores.

En la población evaluada, las enfermedades más prevalentes como motivo de la hospitalización de los ancianos fueron las infecciones, que también constituyeron complicaciones adquiridas durante la estancia en los servicios de salud; de hecho, la infección hospitalaria fue la complicación más frecuente (3,25%) en los pacientes analizados y representó el 46,15% del total de las complicaciones reportadas. Esta cifra es similar a la reportada en el estudio de Li, *et al.*, en el cual se analizaron los factores de riesgo y las estrategias de prevención de las infecciones hospitalarias en ancianos en una población canadiense y se documentó una prevalencia de 3,3% de infecciones adquiridas durante la hospitalización ^18^. Se ha reportado que las infecciones hospitalarias de mayor prevalencia en ancianos son la urinaria, la respiratoria (neumonía), la de piel y tejidos blandos, la del sitio operatorio, la bacteriemia tardía o las asociadas con el uso de dispositivos médicos. Además, los análisis microbiológicos muestran que los microorganismos más frecuentemente aislados son bacterias Gram negativas ^19^.

La combinación de enfermedades y síndromes geriátricos es un importante predictor de complicaciones durante la estancia hospitalaria en los mayores, pues su presencia refleja una reducción de las reservas funcionales y fisiológicas; también, se asocia con el deterioro funcional y cognitivo, la institucionalización y la mortalidad después del egreso ^15^^,^^20^^,^^21^. En su estudio, Hoogerduijn, *et al.,* reportaron que el 83% de los pacientes presentaba deterioro en las actividades instrumentales de la vida diaria; 61%, polifarmacia; 40%, demencia, y 52%, desnutrición, resultados similares a los del presente estudio, en el cual la malnutrición, la demencia, la polifarmacia y la dependencia funcional registraron prevalencias similares ^22^. Este hallazgo es de gran importancia, ya que el reconocimiento temprano de dichos síndromes geriátricos a partir de una valoración geriátrica integral podría reducir los eventos adversos durante y después de la estancia hospitalaria.

La prevalencia de malnutrición entre las personas de edad avanzada en entornos hospitalarios es común, con un rango general de 11 a 45% ^13^. En el presente estudio, los resultados del análisis multivariado determinaron que la malnutrición fue uno de los factores independientes con significación estadística lo que se explica porque una escasa reserva nutricional determina una reacción inmunitaria inadecuada, déficit de factores de coagulación, inadecuadas reacciones metabólicas, retraso en la cicatrización y atrofia muscular ^2^^,^^23^.

En algunos estudios recientes se ha reportado que del 25 al 40% de las personas mayores ingresadas en unidades geriátricas de agudos han sido diagnosticadas con demencia por cualquier causa y que esta se asocia con complicaciones hospitalarias ^19^^-^^25^. La demencia fue muy prevalente en nuestra población, lo que se explicaría por el carácter del hospital como centro de referencia para el estudio y manejo de pacientes con deterioro cognitivo. Sin embargo, en los análisis bivariado y multivariado no se encontró una asociación entre la demencia y el riesgo asociado con las complicaciones hospitalarias. En la revisión sistemática de Fogg, *et al.*, el 60% de los pacientes con demencia asociada a síntomas conductuales y psicológicos presentó complicaciones hospitalarias. Asimismo, el deterioro cognitivo demostró ser el factor de riesgo más importante para el desarrollo de incontinencia urinaria y fecal, delirio, caídas, polifarmacia y úlceras por presión ^26^.

Por otra parte, la mortalidad en pacientes con complicaciones durante la hospitalización aumenta en los mayores de 75 años con múltiples enfermedades y síndromes geriátricos asociados ^16^^,^^23^^,^^27^. En el presente estudio, la diferencia entre la mortalidad del grupo con complicaciones hospitalarias y la del grupo sin complicaciones fue de 29,03%. Estos hallazgos se correlacionan con los datos publicados en el estudio de Fogg, *et al.*, en el que la mortalidad en pacientes hospitalizados fluctuó entre el 4,9 y el 24%; al comparar los pacientes con demencia y aquellos que no la tenían en ocho estudios, llegaron a la conclusión de que los que la presentaban tienen un mayor riesgo de muerte, con estimaciones que variaban según el *odds ratio* ajustado (aOR) entre 1,09 (1,03-1,16) y 2,1 (1,0-4,5) ^28^.

La población analizada en este estudio incluyó una cohorte retrospectiva de personas mayores de 65 años y comparó la presencia de factores cognitivos, funcionales y nutricionales determinantes, los cuales se evaluaron con medidas estandarizadas, por lo cual los hallazgos coincidieron con los de estudios previos ^28^^,^^29^. Además, se utilizó la información disponible en la práctica clínica habitual ^30^ mediante la revisión de las historias clínicas y se definieron claramente los criterios de inclusión y exclusión, lo que reduce el riesgo de sesgos estadísticos. También, se contemplaron factores de confusión relevantes según la revisión de estudios previos ^3^^,^^22^^,^^28^ y se hizo un ajuste multivariado para determinar los factores asociados. Hubo algunas limitaciones, no obstante, pues no fue posible caracterizar el tipo de complicaciones hospitalarias que se presentaron y solo se incluyeron pacientes con la enfermedad índice, mas no los sometidos a intervenciones quirúrgicas. Tales limitaciones son frecuentes en los estudios de pacientes mayores hospitalizados por condiciones agudas y podrían explicar el hecho de que las tasas de inclusión fueran más bajas que en otros estudios.

Los hallazgos del presente estudio tienen implicaciones importantes para la atención hospitalaria de pacientes adultos mayores, ya que se encontró que es relevante intervenir de manera enfática condiciones como el deterioro funcional y la malnutrición, como factores asociados a la aparición de complicaciones hospitalarias. Además, una evaluación sistemática de las condiciones geriátricas en el momento de la admisión en el hospital debe resultar en un plan de tratamiento adecuado con objetivos realistas.

En conclusión, las complicaciones hospitalarias que se presentan en la población adulta mayor son muy frecuentes y aumentan el riesgo de mortalidad. En los pacientes mayores de 65 años, factores como la dependencia funcional y la malnutrición son comunes y se asocian con la aparición de complicaciones durante las hospitalizaciones. Asimismo, los síndromes geriátricos son frecuentes entre los pacientes que presentan complicaciones. Teniendo en cuenta esto, se deberían hacer nuevos estudios con perspectivas más amplias para determinar otras condiciones asociadas con la aparición de complicaciones hospitalarias, así como hacer intervenciones preventivas.

## References

[1] Warner JL, Zhang P, Liu J, Alterovitz G (2016). Classification of hospital acquired complications using temporal clinical information from a large electronic health record. J Biomed Inform.

[2] Correia MI, Waitzberg DL (2003). The impact of malnutrition on morbidity, mortality, length of hospital stay and costs evaluated through a multivariate model analysis. Clin Nutr.

[3] Lamont CT, Sampson S, Matthias R, Kane R (1983). The outcome of hospitalization for acute illness in the elderly. J Am Geriatr Soc.

[4] Rao A, Suliman A, Vuik S, Aylin P, Darzi A (2016). Outcomes of dementia: Systematic review and meta-analysis of hospital administrative database studies. Arch Gerontol Geriatr.

[5] Gutierrez WA (2014). Situacion actual del medico geriatra en Colombia. Universitas Medica.

[6] Baztan JJ, Suarez-García FM, López-Arrieta J, Rodríguez-Manas L (2011). Eficiencia de las unidades geriátricas de agudos: metaanálisis de estudios controlados. Rev Esp Geriatr Gerontol.

[7] Lizarbe-Castro MV, Gamarra-Samaniego P, Parodi Garcia JF (2015). Factores de riesgo asociados a complicaciones intrahospitalarias en adultos mayores del Hospital Nacional Edgardo Rebagliati Martins, Lima, 2010. Horizonte Médico (Lima).

[8] Bohlouli B, Tonelli M, Jackson T, Hemmelgam B, Klarenbach S (2016). Risk of hospital-acquired complications in patients with chronic kidney disease. Clin J Am Soc Nephrol.

[9] Brooks SK, Webster RK, Smith LE, Woodland L, Wessely S, Greenberg N (2020). The psychological impact of quarantine and how to reduce it: Rapid review of the evidence. Lancet.

[10] Szlejf C, Farfel J, Curiati J, Couto E, Jacob-Filho W, Azevedo R (2012). Medical adverse events in elderly hospitalized patients: A prospective study. Clinics (Sao Paulo).

[11] Shah S, Vanclay F, Cooper B (1989). Improving the sensitivity of the Barthel Index for stroke rehabilitation. J Epidemiol.

[12] Guigoz Y, Vellas B, Garry PJ (1996). Assessing the nutritional status of the elderly: The Mini Nutritional Assessment as part of the geriatric evaluation. Nutr Rev.

[13] Shuhada NA Aziz A, Mohd NI Teng F, Abdul MR Hamid (2017). Assessing the nutritional status of hospitalized elderly. Clin Interv Aging.

[14] Kadambi S, Soto-Perez-de Celis E, Garg T, Loh KP, Krok-Schoen JL, Battisti NML (2020). Social support for older adults with cancer: Young International Society of Geriatric Oncology review paper. J Geriatr Oncol.

[15] Velilla NM, Abizanda-Soler P, Leocadio Rodríguez-Manas (2020). Valoración de la carga de enfermedad: multimorbilidad, comorbilidad y enfermedad crónica. Tratado de medicina geriátrica.

[16] Masnoon N, Shakib S, Kalisch-Ellett L, Caughey GE (2017). What is polypharmacy? A systematic review of definitions. BMC Geriatr.

[17] Cristofori G, Montoya IL, Jose J, Cortes B, Abizanda-Soler P, Leocadio Rodríguez-Manas (2020). Sindromes geriátricos: aspectos fisiopatológicos, clínicos y terapéuticos comunes. Tratado de medicina geriátrica.

[18] Li Y, Ren L, Zou J (2019). Risk factors and prevention strategies of nosocomial infection in geriatric patients. Can J Infect Dis Med Microbiol.

[19] Avci M, Ozgenc O, Coskuner SA, Olut AI (2012). Hospital acquired infections (HAI) in the elderly: Comparison with the younger patients. Arch Gerontol Geriatr.

[20] McCusker J, Kakuma R, Abrahamowicz M (2002). Predictors of functional decline in hospitalized elderly patients: A systematic review. J Gerontol A Biol Sci Med Sci.

[21] Creditor MC (1993). Hazards of hospitalization of the elderly. Ann Intern Med.

[22] Hoogerduijn JG, Buurman BM, Korevaar JC, Grobbee DE, De Rooij SE, Schuurmans MJ (2012). The prediction of functional decline in older hospitalised patients. Age Ageing.

[23] Vanderwee K, Clays E, Bocquaert I, Gobert M, Folens B, Defloor T (2010). Malnutrition and associated factors in elderly hospital patients: A Belgian cross-sectional, multi-centre study. Clin Nutr.

[24] Briggs R, Dyer A, Nabeel S, Collins R, Doherty J, Coughlan T (2017). Dementia in the acute hospital: The prevalence and clinical outcomes of acutely unwell patients with dementia. QJM.

[25] Mukadam N, Sampson EL (2011). A systematic review of the prevalence, associations and outcomes of dementia in older general hospital inpatients. Int Psychogeriatr.

[26] Mecocci P, von Strauss E, Cherubini A, Ercolani S, Mariani E, Senin U (2005). Cognitive impairment is the major risk factor for development of geriatric syndromes during hospitalization: Results from the GIFA study. Dement Geriatr Cogn Disord.

[27] Fogg C, Meredith P, Bridges J, Gould GP, Griffiths P (2017). The relationship between cognitive impairment, mortality and discharge characteristics in a large cohort of older adults with unscheduled admissions to an acute hospital: A retrospective observational study. Age Ageing.

[28] Fogg C, Griffiths P, Meredith P, Bridges J (2018). Hospital outcomes of older people with cognitive impairment: An integrative review. Int J Geriatr Psychiatry.

[29] Vidan-Astiz MT, Sánchez-García E, Alonso-Armesto M, Montero-Errasquin B, Martinez-de la Casa A, Ortiz FJ (2008). Deterioro funcional durante la hospitalización en ancianos. Beneficios del ingreso en el servicio de geriatría. Rev Esp Geriatr Gerontol.

[30] Rojano-i-Luque X, Sanchez-Ferrin P, Salva A (2016). Complicaciones de la hospitalizacion en personas mayores. Med Clin (Barc).

